# Glucosidase Inhibitors Screening in Microalgae and Cyanobacteria Isolated from the Amazon and Proteomic Analysis of Inhibitor Producing *Synechococcus* sp. GFB01

**DOI:** 10.3390/microorganisms9081593

**Published:** 2021-07-27

**Authors:** Diana Gomes Gradíssimo, Vivian Cássia Oliveira da Silva, Luciana Pereira Xavier, Sidney Vasconcelos do Nascimento, Rafael Borges da Silva Valadares, Silvia Maria Mathes Faustino, Maria Paula Cruz Schneider, Agenor Valadares Santos

**Affiliations:** 1 Laboratory of Biotechnology of Enzymes and Biotransformations, Institute of Biological Sciences, Federal University of Pará, Belém 66075-110, Brazil; vcassi@hotmail.com (V.C.O.d.S.); lpxavier@ufpa.br (L.P.X.); 2 Post Graduation Program in Biotechnology, Institute of Biological Sciences, Universidade Federal do Pará, Augusto Corrêa Street, Guamá, Belém 66075-110, Brazil; 3 Instituto Tecnológico Vale, Belém 66055-090, Brazil; svn_live@hotmail.com (S.V.d.N.); rafael.borges.valadares@itv.org (R.B.d.S.V.); 4 Laboratory of Algae Cultivation and Bioprospecting, Pharmacy Coordination, Marco Zero do Equador Campus, Federal University of Amapá, Juscelino Kubitschek Highway, Km 2, Macapá 68903-419, Brazil; silviamathes@unifap.br; 5 Genomics and Systems Biology Center, Federal University of Pará, Belém 66075-110, Brazil; mariapaulacruzschneider@gmail.com

**Keywords:** glucosidase inhibitors, α-glucosidase, β-glucosidase, proteome, cyanobacteria, *Synechococcus*, nitrogen, stress, bioprospection, Amazonia

## Abstract

Microalgae and cyanobacteria are good sources for prospecting metabolites of biotechnological interest, including glucosidase inhibitors. These inhibitors act on enzymes related to various biochemical processes; they are involved in metabolic diseases, such as diabetes and Gaucher disease, tumors and viral infections, thus, they are interesting hubs for the development of new drugs and therapies. In this work, the screening of 63 environmental samples collected in the Brazilian Amazon found activity against β-glucosidase, of at least 60 min, in 13.85% of the tested extracts, with *Synechococcus* sp. GFB01 showing inhibitory activity of 90.2% for α-glucosidase and 96.9% against β-glucosidase. It was found that the nutritional limitation due to a reduction in the concentration of sodium nitrate, despite not being sufficient to cause changes in cell growth and photosynthetic apparatus, resulted in reduced production of α and β-glucosidase inhibitors and differential protein expression. The proteomic analysis of cyanobacteria isolated from the Amazon is unprecedented, with this being the first work to evaluate the protein expression of *Synechococcus* sp. GFB01 subjected to nutritional stress. This evaluation helps to better understand the metabolic responses of this organism, especially related to the production of inhibitors, adding knowledge to the industrial potential of these cyanobacterial compounds.

## 1. Introduction

Glucosidases are important enzymes for the correct functioning of various physiological systems, as they are involved in biochemical processes such as the degradation of polysaccharides to monosaccharides—the latter being the form absorbable by our body —and they are responsible for the intracellular digestion of lysosomal glycoconjugates and glycoprotein as well as glycolipid biosynthesis from oligosaccharides [1,2]. Glycoconjugates are present on the surface of cells, acting on cell recognition and playing an important role in viral and bacterial infections, in addition to other immune responses; the malfunction of glycoconjugate synthesis can result in immature glycoproteins, which can also affect the solubility of molecules, cause inflammatory processes, and develop tumor cells [3,4,5,6]. Glucosidases cleave sugars in the intestinal tract forming d-glucose, which is absorbed by the intestine, raising blood glycogen levels. The inhibitors therefore act in a competitive manner, preventing the action of these enzymes and avoiding postprandial hyperglycemia [7,8,9].

The competitive nature of most glucosidase inhibitors suggests that molecular conformation and charge play a fundamental role in the effectiveness and selectivity of inhibitors towards the active site of enzymes. Thus, we find inhibitors that are iminosugars, disaccharides, thiosugars, carbon-sugars, and pseudo-amino-sugars; therefore, the carbohydrate metabolism is a good focus in the search for inhibitors [4,10,11]. The inhibition of α-glucosidases is an important action in the treatment of type two diabetes, a disease that afflicts 420 million people worldwide, about 6% of the world population, and whose mortality increased by up to 80% between 2000 and 2019 [12].

Some commercial drugs use this cleavage inhibition mechanism, such as acarbose, a pseudo-amino-sugar with the trade name precose (Bayer, Leverkusen, Germany), miglitol, from different companies, and *N*-butyl-1-deoxinojirimycin, zavesca (Jannsen, Raritan, NJ, USA) and its generic miglustat, used to treat Gaucher disease. The action of inhibitors such as nojirimycin and its *N*-alkylated derivatives has been studied for a long time for its role in the processing of glycoprotein *N*-linked oligosaccharide, a synthesis pathway used by viruses in host cells [6,13], being an interesting target for antiviral therapies [14,15], including HIV [16,17,18]. A reduced derivative of nojirimycin, 1-deoxinojirimycin, has been shown to inhibit the formation of syncytia—a giant, multi-nucleated cell—one of the greatest cytopathological effects caused by HIV replication [1,19]. In addition, α-glucosidase inhibitors that already commercially available can be of good use facing the current pandemic, COVID-19, such as the aforementioned miglustat [20]. Anti-cancer activity has also been observed in these inhibitors [21], with β-glucosidase inhibitors increasing breast cancer sensitivity to chemotherapy [22] and overcoming the resistance of gastric tumors [23].

With occurrence in animals, plants and microorganisms [24,25,26,27], the bioprospection of glucosidase inhibitors in sustainable natural sources was once concentrated on macroalgae [28,29,30] but it is interesting to look for alternatives with cheaper cultivation and that require less area, such as microalgae, including cyanobacteria [31,32,33,34,35,36,37]. The possibility of associating the production of these inhibitors with other metabolites of biotechnological interest produced by cyanobacteria, such as antioxidants [38]; pigments [39], acids and polysaccharides of cosmetic and pharmacological interest [40,41] in addition to joint production with polyhydroxybutyrate (PHB) [42,43] and biofuels [44,45,46], while mitigating CO_2_ [43,44], is yet another advantage of this bacterial phylum.

The potential of cyanobacteria in the food sector is another aspect that makes them a good target in the search for anti-hyperglycemic actives to control diabetes. *Arthrospira platensis*, for example, has antioxidants as well as antimicrobial and α-glucosidase inhibitory activity [47,48]. The Generally Recognized as Safe (GRAS) status and FDA approval of this filamentous cyanobacterium opens possibilities for the application of other species as a nutraceutical as well [49], as an alternative to conventional treatments with a single drug, sometimes in high doses, which implicates in side effects [50]. The use of lower doses of conventional drugs combined with natural products, in addition to the therapeutic benefit of the synergy of the compounds, enables a cheaper treatment, important for medication of continuous use especially in developing countries [2].

The exact biological function of these inhibitors in cyanobacteria is still unknown, with defense against predators being the most discussed action [33,34,35]. The search for cyanobacteria that produce glucosidase inhibitors in environments with different physical and chemical conditions, and therefore, with different ecological relationships, is a good strategy to find biological compounds that are often still unexplored, such as the search for inhibitors in marine cyanobacteria the Baltic Sea [51], tropical environments [37] and flooded regions in the Nile delta [41,42,43,44,45,46,47,48,49,50,51,52].

In this research article, screening of inhibitory activity for β-glucosidase was performed in 63 environmental samples collected from two states of the Brazilian Amazon. Due to the physicochemical characteristics of this biome, and resulting biological interactions, it was expected that there would be production of glycosidase inhibitors as a response to the natural environment of these cyanobacteria and microalgae. We also evaluated the differential proteome of the α and β-glucosidase inhibitor-producing strain, *Synechococcus* sp. GFB01, in response to sodium nitrate reduction in its culture medium, and how this stress influences the production of glucosidase inhibitors and general metabolic response.

## 2. Materials and Methods

### 2.1. Organisms and Culture Conditions

Microalgae and cyanobacteria, all freshwater organisms, were provided by Genomics and Systems Biology Center (UFPA), and were isolated from the Brazilian Amazon from the states of Amapá—samples collected in October 2013—and Tocantins—collected in November of the same year. Both months are part of the Amazonian summer, the period of the year with less rain (Table S1). The 63 environmental samples were cultured for six weeks, in 100 mL of BG-11 medium in 250 mL flasks and incubated at 23 °C, with 16 h of light (3000 lx intensity) and 8 h in the dark. The organisms used in this work were initially identified by morphological characters in Chlorophytas, being *Stigeoclonium*, *Chlorella*, *Monoraphidium,* and Cyanophytas belonging to the genera *Synechococcus*, *Planktothrix*, *Limnothrix*, *Nostoc* and *Merismopedia*. The best results were identified morphologically, with *Synechococcus* sp. GFB01 (SisGen No. A7A712F), from now on called simply GFB01, isolated from the freshwater lagoon Lagoa dos Índios, Amapá (00°1′52.9248″ N, 51°6′9.2118″ W), molecularly identified, with its 16 s mRNA gene being compatible with the genus *Synechococcus* (98% identity) [53].

The cell growth of GFB01, grown in BG-11 medium containing 1.5 g/L NaNO_3_ and medium with 0 and 0.15 g/L of this nutrient, was monitored, measuring the optical density of the culture, OD_750nm_ [54] during a 30-day period, in triplicate. The chlorophyll α content, in µg/mL, was monitored throughout the cultivation using 1 mL of the culture, to which 1 mL of methanol was added and then read at 663 nm in a spectrophotometer using the formula C (µg/mL) = OD_663nm_ × 12.7 [55,56].

### 2.2. Extraction and Fraction of Intracellular Inhibitors

For the screening of intracellular β-glucosidase, inhibitors were obtained by breaking the cells in deionized water with the use of point ultrasound, with three cycles of 10 s at medium power, in ice bath, obtaining the crude extract [57,58]. For the best result found, GFB01, the aqueous and methanolic fractions were also tested [31,58], adding 10% (*w*/*v*) of resin to the crude extract hydrophobic (Diaion HP-20 by Aldrick), shaking moderately for 30 min at room temperature. Then, the mixture was poured into a glass column and washed with an equal volume of deionized water and then a volume of 100% methanol. The eluted fraction was then evaporated in a water bath at 50 °C to obtain the final organic extract, and the fractions were concentrated in a lyophilizer for the microplate assay.

### 2.3. Screening in Agar Plates

This assay consisted of the preparation of petri dishes containing 2% agar (*w*/*v*), 0.1 M sodium acetate buffer solution at pH 5.0, iron chloride III 5% (*w*/*v*) and esculin 0, 2% (*w*/*v*) [58,59]. The reaction mixture was prepared, adding 1 U of 5.2 U/mg almond β-glucosidase in 0.1 M sodium acetate buffer at pH 5.0, and the extracts, fractions and 0.1 M sodium acetate buffer at pH 5.0. The reaction mixture was incubated at 25 °C for one hour to allow the formation of the enzyme-inhibitor complex. After incubation, an aliquot was added to wells made in the Agar plates. The inhibitory activity was visually verified by the non-formation of dark brown halos, and the activity was monitored for 60 min. If there was no halo formation or it did not form for at least 30 min, the sample was considered as positive for the presence of the inhibitor. As a positive control for inhibition, the irreversible inhibitor conduritol β-epoxide was used, with the negative control consisting only of the enzyme.

### 2.4. Inhibitory Potential Assay for α and β-Glucosidase in Microplate

The inhibitory potential assay was tested against α and β-glucosidase in order to verify if there was a greater specificity between the glucosidase inhibitors produced by GBF01, as well as if the decrease in the concentration of nitrogen in the culture medium affects the production of the inhibitors in a similar way. It followed the protocols of Cannel et al. [31] and Shinde et al. [60], with modifications, for α-glucosidase, and the method of Kaur et al. [61] for the inhibitory potential assay for β-glucosidase. Phosphate buffer 50 mM pH7 was used for α-glucosidase, and acetate buffer 50 Mm pH 5 for β-glucosidase, and the respective substrates, p-nitrophenyl-α-d-glycopyranoside (pNPG-α) and p-nitrophenyl-β-d-glycopyranoside (pNPG-β), with absorbance reading at 410 nm in a spectrophotometer [62]. The inhibition activity was calculates according to the formula [39]:

$$\%\;inhibition=\left(\frac{A_{control}-A_{sample}}{A_{control}}\right)\times 100$$

### 2.5. Search for Biosynthetic Enzymes of Glucosidase Inhibitors

The biosynthetic pathway of the glucosidase inhibitors, nojirimycin and its derivative 1-deoxynojirimycin, two iminosugars, in *Bacillus* sp. was used as reference [63,64,65], in which the enzymes coded by GabT1, Yktc1 and GutB act. The search in Blastp used proteins from the *Bacillus* sp. biosynthetic pathway available in the NCBI as query, and as subject the genome of *Synechococcus* sp. KORDI-100, also available in NCBI, was used as well as the proteome of GFB01 from the current research [53]; proteins with bit score > 50 and E-values < 10^−10^ were considered homologous [66]. For GabtT1, the sequences used as query were WP_007408029; WP_013350833; WP_014304227 and WP_015416683, for Yktc1, as most of the sequences available were from hypothetical proteins, the search used a higher number of the sequences as query, WP_007408028; WP_011996207; WP_013350834; WP_014304228; WP_014416764; WP_015416684; WP_020955231 and WP_022552493. For last step in *Bacillus* nojirimycin biosynthesis, GutB, the following sequences were used as query: WP_003156784; WP_007408027; WP_011996208; WP_013350835 and WP_020955232.

### 2.6. Protein Extraction and Peptide Digestion

To the cell concentrate, 5 mL of lysis solution containing β-mercaptoethanol and 10 µL of protease inhibitor cocktail (Sigma-Aldrich, St. Louis, MO, USA) were added. A sonicator was used and after dissolving the cell concentrate, the sample was separated into 2 mL eppendorf type zip-lock tubes, to which 700 µL of saturated phenol (pH 8) were added. It was centrifuged at 16,000× *g* for 7 min at 4 °C, and this step was repeated for better removal of SDS and other residues. 0.1 M ammonium acetate was added, and dissolved in 100% methanol and then incubated at −20 °C overnight [67].

After precipitation the samples were centrifuged again (16,000× *g*, 4 min at 4 °C), and washed three times with 80% cold acetone and once with 70% ethanol. The pellet was dried and the cell concentrate solubilized in a solution of 7 M urea and 2 M thiourea, and samples were quantified in Qubit. 3.5.2. For protein digestion, 8 M urea was added to the sample and the reduction step was performed with 5 mM DTT, incubating at 56 °C for 25 min; for alkylation, 14 mM iodoacetamide was used with incubation for 30 min protected from light; for removal of free IAA, DTT was added again, and the reaction was incubated protected from light. The diluted sample, in the proportion of 1: 5, with 50 mM ammonium bicarbonate solution, 1 mM CaCl_2_ and the trypsin solution (20 ng/µL) were added, allowing digestion to occur for 16 h at 37 °C [68,69,70].

### 2.7. D-UPLC- Mass Spectrometric Analysis

The samples were desalted using C18 column (SepPack 50 ng from Waters Corp., Milford, MA, USA), and the eluted samples concentrated and resuspended in diluted 20 mM ammonium formate (Villén; Gygi 2008). In a Nano Acquity UPLC liquid chromatograph (Waters), the samples were fractionated in two dimensions, the first in an analytical column, XBridge BEH130 5 µm C18 (300 µm × 50 mm) with a 2000 µL per minute flow, and the second in a trap 5 µm C18 column (180 µm × 20 mm) and a BEH130 1.7 µm C18 analytical column (100 µm × 100 mm) at a flow of 400 µL per minute. The attached ESI-Q-ToF Synapt G2S mass spectrometer (Waters) operated in positive mode, and continuous fragmentation (MSE) with the collision energy oscillating between 5 and 40 eV. Mass spectra were acquired within the range of 50 to 1200 Da, with a 0.5 s scan and an interval between scans of 0.1 s. Mass spectra were acquired in automatic mode [70,71].

### 2.8. Data Analysis and Statistics

For statistics, the GraphPad Prism 6 software was used, and the results were expressed as mean ± standard deviation, considering biological triplicates. The significant differences of the samples in the tests of inhibitory potential were analyzed using the ANOVA test, with significance interval *p* < 0.05. The tandem mass spectra were extracted using ProteinLynx Global Server version 3.0.2. (Waters), and the MS/MS samples analyzed using IdentityE (Waters Corp., Milford, MA, USA); iaDBs version: 2.135.2.0) [70], using a local database assembled from 16 complete genomes of *Synechococcus* sp. available at NCBI using genomes of the strains: S. sp. WH 7803, S. sp. WH 8103, S. sp. UTEX 2973, S. sp. PCC 73109, S. sp. PCC 7003, S. sp. PCC 7117, S. sp. PCC 8807, S. sp. SynAce01, S. sp. KORDI-100, S. sp. KORDI-52, S. sp. KORDI-49, S. sp. PCC 6312, S. sp. WH 8102, S. sp. WH 8109, S. sp. PCC 7502, S. sp. RCC307.

The search with IdentityE used mass tolerance of ion fragments of 0.025 Da and a tolerance of precursor ions of 0.1 Da. In the identification of proteins, Scaffold 4.6.1 (Proteome Software Inc., Portland, OR, USA) was used for validation, with peptides with more than 90.0% probability being accepted according to the Peptide Prophet algorithm [72], and proteins with more than 95.0% probability containing at least one identified peptide. For the differentially expressed proteins present in both conditions, a permutation test (t test) was applied with significance greater than 95% (*p* < 0.05). Cutoffs for more expressed proteins were considered as fold change log^2^ ≥ 1 and those less expressed as ≤−1; in this work we discuss only the proteins present in more than one triplicate. For functional annotation, Blast2Go version 5.2.5 was used and the heatmap of the differentially expressed proteins was calculated in software R using the heatmap.2 from the gplots package.

## 3. Results

### 3.1. Screening of β-Glucosidase Inhibitory Activity

**Figure 1:**
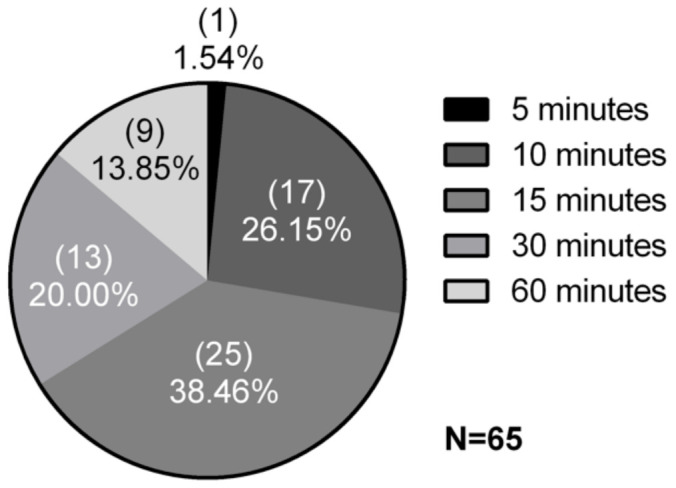
Screening for β-glucosidase inhibition. The 65 extracts tested belong to 63 environmental isolates. Results according to time of inhibition of the enzymatic reaction of β-glucosidase with esculin. Samples with at least 30 min of inhibition were considered positive for the presence of β-glucosidase inhibitors.

As an initial screening, the inhibitory activity against β-glucosidase of 65 samples was tested, corresponding to 63 environmental samples; a GFB01 had its inhibitory action tested using three extracts of different chemical natures: crude—as with the other 62 samples—aqueous and methanolic. The samples were evaluated against the commercial β-glucosidase in an agar plate test. In numerical terms (Figure 1), of the 65 total samples, 33.85% inhibited the enzyme reaction for at least 30 min, being considered positive for the presence of inhibitors. Among the inhibitor-producing environmental isolates, most originate from collection points in environments with anthropization (Table S1) such as rice plantations, Palma’s shore—capital of the state of Tocantins—and Igarapé da Fortaleza. The latter is an area of considerable impact because of the high traffic of small boats to a fair and other points of commerce nearby.

The best result, inhibition of up to 60 min, was observed in nine samples, or 13.85% (Figure S1A); of these, five are microalgae, *Monoraphidium* sp. and four isolates of *Chlorella* sp., and three cyanobacteria, *Synechococcusp* sp. and two strains of *Limnothrix* sp. (Figure S1B). The occurrence of inhibitors in four *Chlorella* sp. samples is indicative of the biotechnological potential of these robust freshwater microalgae, deserving attention for further investigation. Three of the samples originate from the same collection point, besides GFB01, *Limnothrix* sp., P29, and the Chlorophyta *Monoraphidium* sp. were isolated from Lagoa dos Índios, an environment used both for fishing and recreational purposes. Two of the GFB01 extracts are in this group, the crude exctract and methanolic extract. The aqueous extract of this cyanobacterium inhibited the reaction for only 30 min, indicating the methanolic extract as the most suitable for the search for glucosidase inhibitors in this species.

### 3.2. Growth Curve, Chlorophyll a and Protein Content

**Figure 2:**
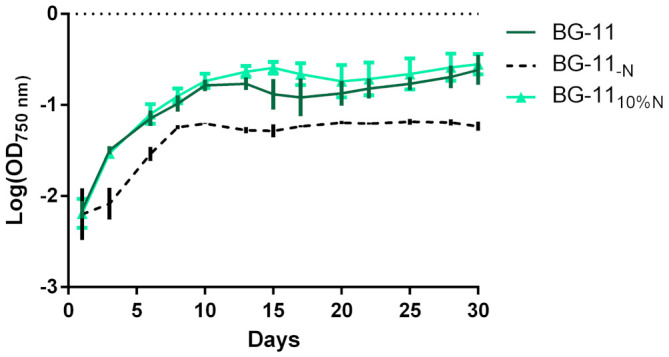
Growth curve of *Synechococcus* sp. GFB01. 30-day cultivation expressed in terms of absorbance at 750 nm, in log. The culture media tested had 1.5, 0.15 and 0 g/L of sodium nitrate.

Due to its good inhibitory activity and sequenced genome being available, GFB01 was selected for further investigation. GFB01 was grown in conventional BG-11 medium and in medium with nutritional stress, reducing sodium nitrate supplementation to 10% of the typical concentration in BG-11 medium (Figure 2). This stress aimed at verifying the response of this cyanobacterium in terms of inhibitor production, in view of the importance of nitrogen in the chemical structure of many glucosidase inhibitors, especially in iminosugars [11].

The depletion of sodium nitrate was detrimental to GFB01, impairing its growth. The chlorophyll α content in the samples with sodium nitrate was similar, 406.69 and BG-11_10% N_ with 395.32 µg/mL of chlorophyll α (Table 1), against 50.52 µg/mL in medium without nitrate, these two were stipulated conditions for the test of inhibitory potential as well as proteomic analysis. Day 15 of each sample, BG-11 and BG-11_10%N_, was chosen for proteomic analysis, due to the higher quantification of proteins in the two samples.

### 3.3. Colorimetric Inhibition Assay for α and β-Glucosidase

**Figure 3:**
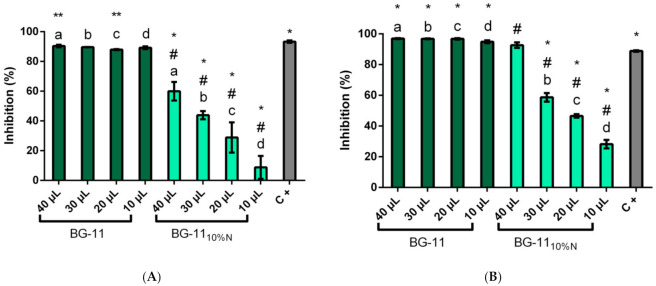
Inhibitory activity for (**A**) α-glucosidase and (**B**) β-glucosidase. In reaction with pNPG-α and pNPG-β, respectively, here shown in %, according to the absorbance at 410 nm in the colorimetric assay, methanolic extracts from *Synechococcus* sp. GFB01 growth in BG-11 and BG-11_10% N_ were evaluated, using conduritol β-epoxide as positive control (C+). (*) Significant difference (*p* < 0.05) between samples and positive control for inhibition, commercial inhibitor; (**) significant difference between BG-11 40 and 20 µL extract, for α-glucosidase; (#) significant difference between BG-11_10%N_ extracts; (a) significance between the different volumes tested, 40 µL; (b) 30 µL; (c) 20 µL and (d) 10 µL.

In the α-glucosidase inhibition assay for the methanolic extract (Figure 3), the enzyme was constantly inhibited, with a significant difference (*p* > 0.05) only between the sample with 40 µL, which showed the highest inhibitory potential, with average inhibition of 90.36 ± 0.82% and sample with 20 µL of extract, with average of 87.97 ± 0.36%. The positive control, conduritol β-epoxide, presented 93.33 ± 0.81%, similar to the inhibitory activity in the methanolic samples tested.

The BG-11_10%N_ extracts showed less inhibitory activity against α-glucosidase and a dose-dependent behavior, with the highest inhibition observed in the sample with 40 µL of extract, with a 59.97% ± 6.25% inhibition and only 8.75% ± 7.71% with 10 µL. The decrease in the inhibitory action in cultivation with nutritional stress indicates that the reduction of nitrogen affects the production of inhibitors by GFB01.

As for the inhibition of commercial β-glucosidase, the extracts cultured in BG-11 showed no significant difference between the four volumes of extract tested, with an average inhibition of 96.32% ± 0.98%, a higher inhibitory activity than that of commercial standard, 88.84% ± 0.49%. Again, the medium with nutritional stress was less efficient in the production of inhibitors, although maintaining good activity, presenting a dose response pattern; in this condition the greatest inhibition was found in the sample with 40µL, 92.63% ± 1.79%, superior to conduritol β-epoxide inhibitory activity in this assay.

### 3.4. Potential Biosynthetic Enzymes

Of the enzymes in the pathway, only the one responsible for the amination step, GabT1, resulted in homologous proteins in the genome of *Synechococcus* sp. KORDI-100 and in the draft genome of GFB01, using *Bacillus amyloliquefaciens* WP_014304227 protein as query (Table 2). The four sequences identified in the current work, both in BG-11 and BG-11_10%N_, also showed homology with the other three queries, WP_007408029 and WP_015416683 of *Bacillus* sp. and WP_013350833 for *Bacillus subtilis*, all with bit scores between 97.8 and 100 and E-values < 10^−10^. In *Synechococcus* sp. KORDI-100 sequence WP_038545752 also showed homology with the other queries from GabT1, with bit scores of 138 and 139. However, as with GFB01, the other biosynthetic enzymes also showed no homology with sequences of these cyanobacteria.

Regarding the dephosphorylation step, mediated by Yktc1, corresponding to a phosphatase or kinase, we did not detect in the differential proteome of GFB01 any sequence with bit score >50, so none was considered homologous with any of the eight query sequences in *Bacillus* sp. In addition, no homologous sequences to GutB1 which is responsible for the dehydration of the compound 2-amino-2-deoxy-D-mannitol to mannojirimycin in *Bacillus* sp., were identified; this compound is a mannosidase inhibitor typically synthesized from chlorobenzenes [63,64].

### 3.5. Protein Profile of Synechococcus sp. GFB01 Subjected to Nutritional Stress

**Figure 4:**
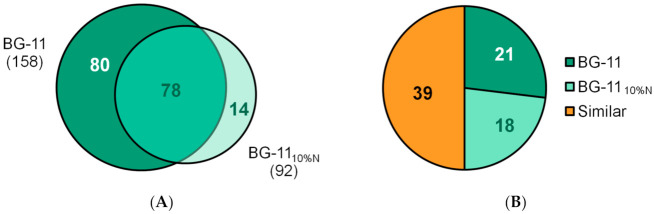
Protein detection profile. (**A**) Venn diagram of proteins identified exclusively in BG-11 and BG-11_10% N_ and in both conditions (intersection); (**B**) proteins up-regulated in each sample, as well as those with similar expression.

Considering only the proteins detected in more than one triplicate among the groups, we have 172 identified and quantified proteins (Figure 4A). Of these, 80 are present exclusively in the control sample, BG-11, and 14 exclusively in the sample subjected to nutritional stress, 78 are present in both conditions, 21 of which showed higher detection in BG-11, while 18 were up-regulated in BG-11_10%N_ and 39 proteins showed similar detection in both conditions (Figure 4B). The list of proteins found in BG-11 and BG-11_10%N_, both exclusive and common among them, is shown in Table S2.

### 3.6. Differentially Expressed Proteins

**Figure 5:**
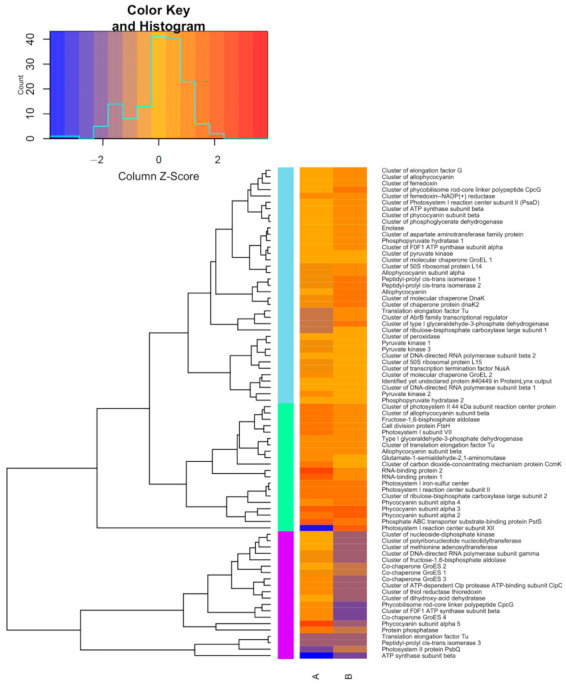
Heatmap with hierarchical grouping of average differential expression of proteins common to both conditions, with A being the culture in BG-11 medium, and B in BG-11_10%N_. Under-expressed proteins are shown in blue hues, over-expressed in red, and orange for similar expression in the two conditions.

Proteins common to both conditions were grouped hierarchically according to the accumulation intensity (Figure 5), using the average in each medium, with A being the complete BG-11 medium, and B the medium with nutritional stress. The purple group contains the least expressed proteins among the 78 common proteins in both conditions, most of them in the stressed sample. Among them, proteins from phycobilisomes and energy metabolism, ATP synthase, also under expressed in BG-11, and fructose 1,6-bisphosphate aldolase.

In this group we also find four chaperones and a thiol reductase, related to the stress response. These environmental stress protection proteins showed greater expression in conventional BG-11 medium, where we also have an up-regulated phosphatase and a phycocyanin subunit. Still in the purple group, the control culture showed less expression of PsbQ protein from photosystem II, while the stress medium accumulated this protein essential for achieving greater activity and stability of this metabolic process. An isomerase and a Tu elongation factor showed similar expression in both conditions.

Another Tu elongation factor was also less expressed in BG-11, as well as a transcriptional regulator from the AbB family, a Rubisco subunit and a glyceraldehyde-3-phosphate dehydrogenase, which was more expressed in stressed culture. These proteins integrate the light blue group where we find the chaperones, GroEL and Dnak, which, unlike the aforementioned chaperones, were up-regulated in response to nutritional stress. Still on stress protection proteins, we found in this group other ferredoxins, these with greater expression in BG-11_10%N_. The light green group shows proteins up-regulated in both conditions, mostly with higher expression in the control condition. These include proteins that are essential for the cell function and thus, the cyanobacteria survival, such as ribosomal proteins for de novo synthesis, glycolysis, photosynthesis and cell respiration.

### 3.7. Proteins in Metabolic Pathways

**Figure 6:**
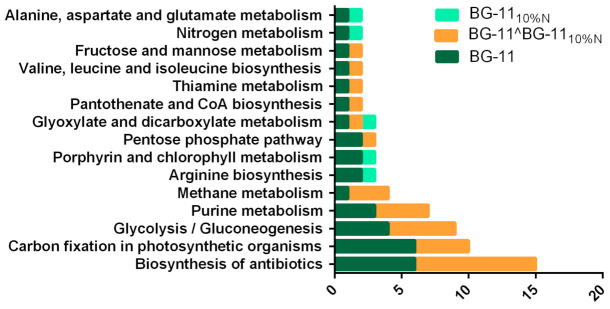
Protein sequences in the two culture media, exclusive and common, attributed to metabolic pathways. According to Blast2GO annotation into KEGG pathways, here are shown pathways with more than one sequence attributed.

The protein sequences found participate in 26 metabolic pathways (Figure 6, pathways with more than one sequence), with 76 sequences in BG-11 integrating 23 pathways, 4 of them found exclusively in this condition. For BG-11_10%N_ we have 19 metabolic pathways. It is important to note that the functional annotation can assign the same sequence to more than one protein and, consequently, to more than one pathway; this is the case with the sequences WP_015168853 and WP_011933362 in BG-11_10%N_, both of which are a synthase (EC: 6.3.1.2) with action on nitrogen, arginine and alanine, aspartate and glutamate metabolisms.

**Figure 7:**
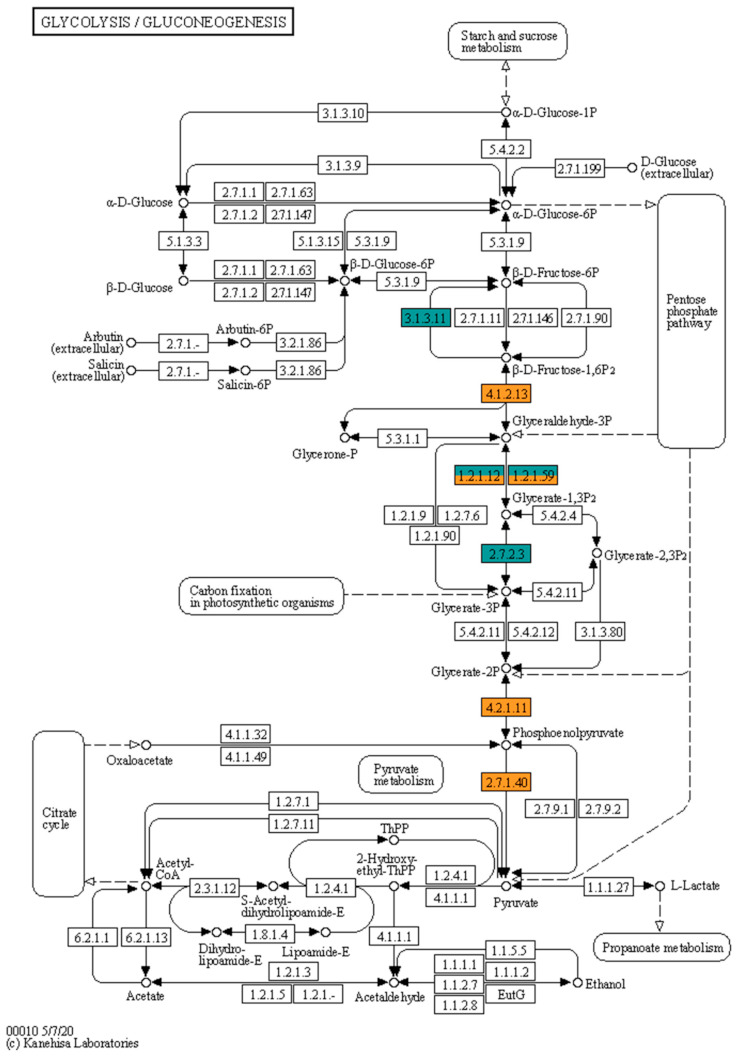
Molecular response in the glycolysis/gluconeogenesis pathway of *Synechococcus* sp. GFB01 cultured in BG-11 and BG-11_10%N_. In orange are enzymes expressed in both conditions, and in dark green BG-11 exclusive proteins. Proteins EC: 1.2.1.12 and EC: 1.2.1.59 were detected as five sequences not shared by the two conditions tested, three in BG-11. Image based on the KEGG database.

The largest number of sequences was attributed to antibiotic biosynthesis, with six of them exclusively in BG-11, the same number of exclusive sequences in this medium for carbon fixation, which in total presented 10 sequences. Glycolysis and gluconeogenesis (Figure 7) are of interest for their role in the processing of sugars, in this group of the 16 sequences attributed to 9 metabolism proteins, 4 enzymes, or 5 sequences, are present exclusively in the medium of greater production of inhibitors. In the metabolic map we have seven enzymes detected in the present work, with the dehydrogenases (EC: 1.2.1.12 and EC: 1.2.1.59) from the conversion step from d-glyceraldehyde 3-phosphate to 3-phospho-d-glyceroyl phosphate assigned to five sequences, three being exclusive to the BG-11 medium and the other two detected in both conditions, one with similar expression and the other over expressed under stress (Log_2_ FC of 2.85).

## 4. Discussion

### 4.1. Growth Curve: Influence of Nitrate Concentration

Cultures in complete medium and in reduced nitrate showed similar growth (Figure 2). The nitrate-free medium proved to be harmful to the strain, as, since the *Synechococcus* genus does not have nitrogenase, it cannot fix nitrogen from the environment; however, this species has the advantage of being able to use nitrates and nitrites from the environment as a nitrogen source [73], this being due to two genes responsible for the expression of nitrate and nitrite reductase, narB and nirA, respectively [74]. The positive relationship between greater availability of nitrate and higher growth is well reported in *Synechococcus* sp. [75], as long as it is within the biologically relevant concentrations [76]. In this study, the reduction of nitrate did not hinder the growth of the strain, which showed growth similar to the complete medium with a slight drop in the production of chlorophyll α, indicating that the stress may not have been enough to alter the growth and production of biomass.

With an exponential phase from the third to the twentieth day, then passing to the stationary phase, this strain showed slower growth when compared with data from the literature for this genus of coccoid, such as *Synechococcus* sp. BO 8807 and *Synechococcus rubescens* SAG, that reach the stationary phase with about 13 days [77]. In *Synechococcus* sp. CACIAM66, another species isolated from the Amazon environment, exponential growth was observed until the 17th, closer to that observed in the current work, but showing a decrease on the 20th day [78].

### 4.2. Inhibitory Potential of the Methanolic Extract of Synechococcus sp. GFB01

The presence of glucosidase inhibitors has already been reported in microalgae and cyanobacteria, such as the screening carried out with 500 samples of fresh water and the marine environment, where 6 samples of fresh water cyanobacteria, all grown in BG-11, showed inhibition of α-glucosidase between 85% and 100% [31], with aqueous and methanolic extracts being the most employed in the work as a whole. The extraction method is decisive in the selection of inhibitory compounds, as well as their concentration, the butanol extract of *Arthrospira platensis*, for example, had its activity enhanced from IC_50_ of 23 µg/mL to 145 µg/mL [47] in the hexane fraction; thus, the low inhibitory activity of some samples may come from the tested extract.

The present work found inhibition of 90.4% of α-glucosidase in the highest concentration of methanolic extract from cultivation in complete medium (Figure 3A); this activity dropped to 60% in the same concentration, with the strain grown in medium with nitrogen limitation reaching only 8.8% of inhibition in the concentration of 10 µL of the extract, with the lowest concentration of BG-11 inhibiting 89.2% of α-glucosidase. The lower inhibitory activity in nutritional limitation may be related to the role of nitrogen in the chemical structure of glucosidase inhibitors, where a nitrogen atom is almost always present in the anomeric center of the structures of effective glucosidase inhibitors. The interaction at the active site of hydrogen bonds with a carboxylic acid group, which intensifies the positive charge of the anomeric center, shifting the charges to the ring with four nitrogen atoms, favors interactions with the carboxylate group of glucosidase; therefore, inhibitors with one or more nitrogen atom, this one adjacent to the oxygen of the *O*-glycosidic bond, have shown greater anti-β-glucosidase selectivity [11].

The potential anti-hyperglycemic action of cyanobacteria has also been attributed to their pigments, evaluating the inhibitory activity of α-glucosidase in crude extracts, digested extracts—simulating the metabolization of extracts in the human body—and isolated pigments of *Lyngbya*, *Microcoleus* and *Synechocystis* sp. [39]. However, unlike what was observed in GFB01, the crude extract was not as active against the enzyme, with a maximum inhibitory action of 62% for the *Lyngbya* extract. *Synechocystis* sp., closest phylogenetically to GFB01, only showed inhibitory activity in the digested extract, inhibiting 92% of commercial α-glucosidase. The isolated pigments of this species, lycopene and myxoxanthophyll, after digestion, inhibited 93% and 85% of α-glucosidase, with the greatest inhibition of α-glucosidase in this study, 96.6%, achieved by the purified C-phycoerythrin obtained from the two filamentous species. More recent in silico studies corroborate with these findings, pointing to the affinity of the enzyme α-glucosidase for the phycocyanin of cyanobacteria [79].

Other α-glucosidase inhibitory compounds identified in cyanobacteria are present in biofilms [32], including the extra and intracellular polysaccharides [37], the latter with action well below that observed in methanolic extracts, with the greatest anti-α-glucosidase action, 14%, detected in extracellular polysaccharides from *Pseudanabaena* sp., in *Synechococcus* sp.; these polysaccharides inhibited 3% of α-glucosidase, suggesting that the inhibitory activity of GFB01 might have another origin.

The methanolic extract of GFB01 was also active against β-glucosidase (Figure 3B), reaching 96.9% inhibition in BG-11, at the highest concentration of the extract. Although the literature places nitrogen as most significant for β-glucosidase inhibition [11], the results show that the production of α-glucosidase inhibitors was more affected by nutritional stress. When grown in nitrate reduction medium, GFB01 inhibited up to 92.6% of the β-glucosidase enzyme, higher than the best result for α-glucosidase. This inhibitory action of GFB01 against β-glucosidase as well is interesting, since many studies deal with the search for α-glucosidase inhibitors, with the allelopathic action of microalgae being attributed to this action [36,80].

More recent studies have searched for β-glucosidase inhibitors, especially in the marine environment; in a screening with 27 cyanobacteria, 21 showed anti-α-glucosidase action and 22 against β-glucosidase, three of which were active against the two enzymes, *Pseudanabaena* cf. *galeata*, *Nodularia spumigena* and *Synechocystis salina* [51]. An inhibitor with action for both enzymes, already identified in the filamentous genus *Cylindrospermum*, DMDP (2(R),5(R)-bis-(hydroxymethyl)-3 (R),4(R)-dihydroxypyrrolidine), unlike what was observed in GFB01 methanolic extract, showed greater action against α-glucosidase, with an IC_50_ of 84 ±6.8 nM against 201 ± 6.4 nM for β-glucosidase proving to be effective especially against digestive α-glucosidases, with an action superior to 1-deoxynojirimycin [34]. The analysis of the composition of the methanolic extract of *Oscilatoria acuminata* attested the presence of eicosanoic acid, an α-glucosidase inhibitor originating from arachidonic acid [33,41], whose metabolic pathway of unsaturated fatty acids was not identified in GFB01. Anti-α-glucosidase peptides detected in silico in *Arthospira platensis* [48] were also absent in the present study; thus, the investigation of the inhibitory action of the methanolic extract of GFB01 focused on proteins mainly related to phycobilisomes and phycocyanins [39,79] and carbohydrate metabolism, origin of DMDP and nojirimycin [81,82].

Although no occurrence of nojirimycin in cyanobacteria has been reported, its biosynthesis in plants being related to the production of DMDP, this polyhydroxypyrrolidine synthesized from fructose undergoes transamination, oxidation and cyclization reactions. In [81], the nojirimycin biosynthesis enzymes in *Bacillus* sp. were the focus of our search [63,64,65]. The detected proteins are part of the family of aspartate aminotransferases, an action corresponding to GabT1 in the biosynthetic pathway in *Bacillus*. According to KEGG, the detected proteins participate in porphyrin and chlorophyll metabolism, related to photosynthetic pigments and heme proteins [83]. The sequence with the best bit score, WP_041430506, was detected exclusively in BG-11, where there was a greater production of inhibitors. WP_015167166.1 is also exclusive to this condition, and both proteins are annotated as glutamate-1-semialdehyde 2,1-aminomutase (EC: 5.4.3.8), their action also attributed to the sequences common to both BG-11 and BG-11_10%N_, expressed with similar intensity, WP_051847317 and WP_071802316.1. These four proteins act in photosynthesis metabolism and carbon fixation in photosynthetic organisms, which, as with carbohydrate metabolism, was more active in stress-free cultivation, with 22 exclusive proteins, out of 56 total in this group, against 3 exclusive in BG-11_10% N_, which may be related to the increased production of pigments with inhibitory potential in complete medium, since phycocyanobilin is related to carbohydrate metabolism enzymes [79].

In terms of the occurrence of phycocyanins identified in the present work, of the 13 phycocyanins, and their subunits, detected, two sequences are exclusive to the complete medium and 11 are expressed in both conditions, three with similar expression, four over expressed in the control and four in BG-11_10%N_. Despite this balanced division, the BG-11 medium contains proteins related to the production of pigments that were not detected in the nutritional limitation, including cytochrome proteins, carbon dioxide concentration—which favors the accumulation of carbohydrates [54]—and, differently from what was observed, it tends to present greater expression in cyanobacteria under stress [84], in addition to the aforementioned aminotransferases.

### 4.3. Differential Proteome of Synechococcus sp. GFB01: Stress Response

Although abiotic stress, whether due to salinity [85], light cycles [54,86], heavy metals [87] or even nutritional stress [88], can increase protein expression, by activating alternative pathways in the face of metabolic imbalance [69,89,90], in the present work the reduction of nitrate in the culture medium, despite not significantly influencing the growth of the strain or the production of Chlorophyll α (Table 1), and even presenting a higher protein content on day 15—with 693 μg/mL under stress against 546 μg/mL in BG- 11—resulted in the identification of fewer proteins, with only 14 exclusive to this condition (Figure 4A). The correlation of higher protein expression with nitrate availability has also been observed in *Synechococcus* sp. [75], with the nitrogen source also affecting this parameter [91].

Regarding the proteins present in the two conditions (Figure 5), there was under expression of GroES co-chaperones in the medium with nutritional stress, heat shock-related protein [85,92]; this class of proteins is also reported in saline stress [93], and these proteins have a role in the maintenance of cytoplasmic and thylakoid proteins [94], which may be related to the lower amount of proteins identified under stress. This stress also resulted in the greater expression of some photosystem proteins, except for a 44 kDa cluster of photosystem II subunit reaction center protein and Photosystem I subunit VII protein which showed greater expression in BG-11, and Photosystem I reaction center subunit II and Photosystem I iron-sulfur center with similar expression. Some of these proteins were found with greater expression in *Synechocystis* sp. PCC 6803 hrcA mutant [95], more sensitive to heat shock, the exception being a 13 kDa Photosystem II protein and Ferredoxin, over expressed in the mutant. In GFB01 a ferredoxin and ferredoxin-NADP(+) reductase cluster were over expressed in BG-11_10%N_, and heat shock-response proteins Dnak and dnak2 also had greater expression in this condition, as observed in *Synechococcus* PCC 7942 with nitrogen deprivation and in *Synechocystis* sp. PCC 6803 in heterotrophy.

Another protein that showed greater expression in nutritional limitation, with under expression in the control, is a glyceraldehyde-3-phosphate dehydrogenase, and in this group there is also a glyceraldehyde-3-phosphate dehydrogenase with similar expression in both conditions; this protein is associated with carbon fixation [96] and is essential to the Calvin cycle in the catabolic degradation of glucose [97,98]. It is an important biotechnological hub for the production of Isopentenyl pyrophosphate (IPP) and 4-Dimethylaminopyridine (DMAP) for terpenes biosynthesis, 3-hydroxypropionic acid [99] and lipids [100] including PHB [42]. The limitation of nitrogen seems to increase its expression, with the reduction of nitrate increasing the production of lipids in *Synechocystis* sp. CACIAM05 [45,46,47,48,49,50,51,52,53,54,55,56,57,58,59,60,61,62,63,64,65,66,67,68,69,70,71,72,73,74,75,76,77,78]. Moreover, the influence of nitrogen on the production of volatile compounds by GFB01 has already been investigated [101]. In addition to its importance in the production of secondary metabolites, the glyceraldehyde-3-phosphate dehydrogenase-CP12-phosphoribulokinase complex, mediated by thioredoxin, proved to be protective of the enzymes of the Calvin cycle against oxidative stress [102]. In addition to the glyceraldehyde-3-phosphate dehydrogenase common to the two conditions, three others were identified exclusively in the control condition, which overall showed more proteins from carbohydrate metabolism. This finding may be related to the existence of two of these enzymes in cyanobacteria with divergent function, Gap1, acting on glycolysis and Gap2 in the cycle and photosynthetic Calvin [98].

The lower expression of thioredoxin, as well as other stress proteins, in BG-11_10%N_ can be a sign of poor adaptation of the strain to stress, since these proteins are a response to oxidative stress [103] and have been shown to act on the transport of photosynthetic electrons and the assimilation of carbon and nitrogen [69,104]. Thioredoxin was significantly overexpressed in *Synechococcus* sp. WH8102 with cultivation with nitrate and urea [91], and was also detected in *Synechocystis* sp. PCC 6803 acclimated to saline stress [85], where there was an accumulation of carbohydrate metabolism proteins, including glycolysis and Calvin cycle proteins, metabolisms conditioned to the potential redox cell mediated by thioredoxin [104].

### 4.4. Changes in Metabolic Pathways in Response to Nutritional Limitation

As for the proteins involved in metabolic processes, 61 of the 172 total sequences were assigned to KEGG pathways by Blast2GO automatic annotation (Figure 6). Then, performing manual curation, the proteins were classified into eight groups according to their function and gene ontology terms (Table S2). The group with the most proteins is the photosynthesis and carbon fixation with 56 proteins; this metabolism was more active in the complete medium with 53 proteins in this condition against 34 in stress, behavior verified in other cyanobacteria [105,106,107,108]. Among the 31 proteins common to the two conditions, we have two CpcG, one over expressed in BG-11_10%N_ and one under expressed in this condition, and this polypeptide linked to phycobilisome had its expression maintained in *Synechocystis* sp. PCC 6803 [84] under nitrogen starvation, reducing its expression with the replenishment of the nutrient. The same behavior was observed in *Arthrospira* sp. PCC 8005 at the transcriptome level [107], when, in Zarrouck’s medium modified with 5% of the conventional nitrogen requirement, CpcG kept its expression while CpcG2 reduced it; this is related to the degradation of phycobilisomes. This divergence between transcripts and expressed proteins was observed in genes related to photosynthesis in general, including phycobilisome linker polypeptides and phycocyanin synthesis, despite the fact that the production of phycocyanins was affected by nutritional stress in *Arthrospira* sp. PCC 8005, falling from 8% to 1.34% of biomass [107].

Another indication of the consumption of protein apparatus to obtain nitrogen under stress is the presence of glutamine synthetase exclusively in BG-11_10%N_, proteins responsible for transferring the amine groups to 2-oxoglutarate [107,109]. Despite this, the small NblA polypeptide responsible for triggering the degradation of phycobilisomes [110,111] was not detected under any of the conditions, possibly because the stress was not enough to lead the organism to chlorosis. An ATP-dependent ClpC was detected in both conditions, under expressed in BG-11_10%N_, this subunit interacts with NblA, functioning as an adapter to the Clp protease complex initiating the phycobilosomes’ degradation process [112]. The ambiguous results in relation to the phycobilosomes’ degradation apparatus, which includes the phycocyanin pigments, of inhibitory action against glucosidases [39,79] may be justified, again, by the milder stress to which GFB01 was submitted, with the reduction of nitrate to 10% of the control condition, instead of total nitrogen deprivation, being detrimental only for some metabolic processes.

In the nitrogen metabolism, the detected proteins are not shared by BG-11 and BG-11_10%N_, indicating different metabolic behavior in the assimilation of nitrogen. Four sequences were assigned as glutamine synthetase (EC: 6.3.1.2), part of nitrogen and arginine metabolism, acting on the conversion of ammonia to l-glutamine, with two of these sequences detected exclusively in nutritional stress, glutamine synthetase—a key enzyme in the donation of molecular nitrogen, catabolizing arginine and aspartate with increased production of glutamate and glutamine [106]—and two exclusive in the control, cluster of type I glutamate-ammonia ligase. In addition to these, three other sequences were detected only in BG-11, nitrate transport ATP-binding subunits—transmembrane protein of active nitrate transport [113]—urease subunit alpha and lysine-tRNA ligase protein involved in acclimation to nitrogen limitation *in Synechococcus* sp. PCC 7942 [88]. This finding diverges with the literature, with urease genes expressed in nutritional stress due to nitrogen limitation, as well as other environmental disturbances. In *Synechocystis* sp. PCC 6803 [69,109]. The same was observed in *Prochlorococcus* [114], with urease being detected only in nitrogen deprivation and absent when cultivated in nitrate and interestingly in cultivation with urea. The C subunit of the nitrate transporter, as well as urease, also had the opposite behavior in *Synechocystis* sp. PCC 6803 when compared to GFB01, presenting over expression under nutritional limitation and reducing its expression with nutrient replenishment [84]. In *Synechocystis* sp. PCC 6803 proteins involved in the assimilation and transport of alternative nitrogen sources, such as nitrate, were under expressed in different nutritional stresses, including the reduction of nitrate [69,105].

The differential expression of nitrogen acquisition proteins in cyanobacteria subjected to nutritional limitation is reported in different species [105,114,115,116,117], being related to the accumulation of carbohydrates in cyanobacteria [118] in the form of glycogen and/or PHB [42,108,119], with stress favoring greater expression of proteins in the glycolysis, oxidative pentose phosphate and glycogen pathways [120], with light exerting a strong influence on this accumulation [121].

Interestingly, in the present study there was a higher expression of proteins from carbohydrate metabolism in the medium without nutritional stress, with 4 proteins from the pathway being found exclusively in the BG-11 medium, in addition to the 11 proteins common to both conditions. The complete medium also presented transketolases that, in addition to participating in carbon fixation, act on the pentose phosphate pathway. There was in GFB01 without stress greater glycolysis activity (Figure 7) with an exclusive fructose 1,6-bisphosphatase (EC: 3.1.3.11) and two fructose-1,6-bisphosphate aldolase sequences (EC: 4.1.2.13) with higher expression in this condition. This step showed reduced activity in *Arthrospira* sp. PCC 8005 [107] in nitrogen limitation with under expression of fructose 1,6-bisphosphatase; however, unlike what was observed in the present study, this filamentous cyanobacteria, which, like GFB01, is non-diazotrophic [122], showed a stimulation of gluconeogenesis, with over expression of phosphoenolpyruvate synthase—not detected in GFB01 but of similar action to pyruvate kinase (EC: 2.7.1.40), attributed to 4 sequences, three with greater expression in BG-11 (Log_2_ FC up to −3.41) and one over expressed in BG-11_10%N_ (1.48)—and enolase (EC: 4.2.1.11) [106,107], function attributed to two sequences of similar expression in both conditions and a phosphopyruvate hydratase of lower expression under stress (Log_2_ FC −1.46). This behavior is attributed to the conversion of proteins into carbohydrates during gluconeogenesis, albeit to a limited extent [107], which does not seem to be happening with GFB01 due to the lower expression of carbohydrate metabolism and the maintenance of cell growth, which has not affected by this stress.

One metabolism that was negatively affected by stress was protein synthesis, with most of the synthesis proteins being found exclusively in BG-11 medium. Down-regulation of these proteins was observed in *Arthrospira* sp. PCC 8005 [106,107] in nitrogen limitation as occurs in *Synechocystis* sp. PCC 6803 [44] and also in this strain subjected to salt stress [85], and in *Synechococcus* sp. PCC 7002 grown in medium A without combined nitrogen sources, where the decrease in protein synthesis favored the Entner–Doudorof glycolytic pathway [108]. Since 2-keto-3-deoxygluconate-6-phosphate was not detected under either condition, GFB01 probably uses the conventional glycolysis pathways, Embden– Meyerhof–Parnas, and oxidative pentose phosphate pathway [123], similar to that found in *Synechococcus elongatus* UTEX 2973 [121].

Although cell growth was not impacted by stress, cell division proteins were more expressed in BG-11 medium, with 20 exclusives to this medium—13 of them FtsH proteins, an important defense against photoinhibition [103,109]—with the exceptions being three proteins exclusively detected in stress, a cluster of SMC chromosomal segregation protein and a cell repair ATPase and a hypothetical protein-annotated as DNA-templated transcription, termination (GO:0006353). Another hypothetical protein exclusive to BG-11_10%N_, WP_065710894, is related to transport, annotated as porin (GO:0015288) acting in the transport of carbohydrates (GO:0008643). An integral membrane hemolysin activation protein (GO:0016021) was also found exclusively under stress; once again, the metabolism of GFB01 in the two growth conditions diverged, with different membrane proteins and porins being expressed exclusively in BG-11. The identification of few transport proteins is reported by other authors in single-celled cyanobacteria [85], with stress, whether saline, nutritional or by heavy metals, increasing the transmembrane transport of proteins [95,105].

The results obtained showed divergences, and at times the opposite behavior, to that observed in other cyanobacteria in the literature, even in phylogenetically close organisms closely and in the *Synechococcus* genus. Such discrepancy may be justified in the environmental origin of GFB01. The ecological niche directly influences the protein expression metabolism of cyanobacteria, with some pathways being more expressed, or even exclusive, in organisms of a certain environment, even in the case of essential metabolism such as glycolysis [123] and nutrients transport, as occurs with ABC-type NrtABCD transporter, also detected in the present study, which occurs only in freshwater organisms [124].

In this sense, more studies with cyanobacteria isolated from the Amazonian environment are necessary, specifically proteomic analysis, in order to allow a more adequate comparison. The higher expression of stress response proteins, commonly over expressed in stress conditions, detected in GB01 grown without nutritional limitation may indicate the adaptation of this strain to environmental pressure in its natural habitat. The Lagoa dos Índios in Amapá, despite being an area of permanent preservation protected by Brazilian legislation—Law no. 0835, from 2004 and Law No. 12,651, from 2012 [125,126]—has been undergoing increasing anthropization, with unplanned urbanization [127], as well as cattle breeding in the vicinity [128], which impacts water quality.

A recent study found an increase in temperature and a decrease in the dissolved oxygen (DO) in this environment between the years of 2008 and 2017 [129], conditions that favor, and can also indicate, bloom formation [129,130]. This potential eutrophication may have acted as a driving force, stimulating the production and selecting organisms that produce glycosidase inhibitors, as an attempt by strains of defense against other organisms, including other cyanobacteria and microalgae. The occurrence of defense genes such as resistance to heavy metals and antibiotics in the genome of GFB01 [53] is another indication of the adaptation of this strain to the environment.

## 5. Conclusions

Amazonian cyanobacteria and microalgae proved to be an interesting focus of research on glucosidase inhibitors, with anti-β-glucosidase action of at least 30 min detected in 22 of 65 extracts, 9 of which were considered more promising for inhibiting the enzymatic reaction for one hour.

One of these, strain *Synechococcus* sp. GFB01, was also active against α-glucosidase, inhibiting up to 90.2% of the enzyme in a microplate assay, with β-glucosidase inhibition reaching 96.9%, displaying reduction of inhibitory activity, mainly for α-glucosidase, when the organism was cultivated in BG-11 with 10% of the initial sodium nitrate concentration, showcasing the importance of nitrogen for the production of inhibitors. Nutritional stress, although milder than other studies in the literature, also altered protein expression, with stress decreasing the activity of metabolisms such as carbohydrates, associated with the production of enzyme inhibitors, and pigment production. Stress response proteins were mostly accumulated in stress-free cultivation, indicating a pre-existing adaptation of this organism to environmental disturbances.

This is, to the best of our knowledge, the first proteomic analysis of a cyanobacterium isolated from the Amazonian biome; therefore, the present work contributes to the development of products of biotechnological interest through better understanding the protein response and production of glucosidase inhibitors aiming at nutritional optimization.

## Figures and Tables

**Table 1:** Chlorophyll α and protein quantification (μg/mL) in *Synechococcus* sp. GFB01. Growth in complete medium and under nutritional stress, at day 15 and 30.

Parameter (μg/mL)	BG-11		BG-11_10%N_	
	Day 15	Day 30	Day 15	Day 30
Chlorophyll α	406,686	980,088	395,319	971,247
Protein	546	573	693	166

**Table 2:** Sequences homologous to *Bacillus amyloliquefaciens* Aspartate aminotransferase protein, coded by GabT1, found in *Synechococcus* sp. GFB01 in the current proteomic analysis.

ASNo. ^a^	Organism	Identity (%)	E-Value	Bit Score	Log_2_ FC ^c^
WP_038545752 ^b^	*S*. sp. KORDI-100	27.612	2.76 × 10^−^^38^	139	-
WP_041430506	*S*. sp. GFB01	26.426	1.73 × 10^−^^42^	101	BG-11 ^d^
WP_071802316		27.711	1.49 × 10^−^^25^	100	−0.97
WP_051847317		24.750	3.57 × 10^−^^25^	99.8	0.56
WP_015167166		25	1.03 × 10^−^^24^	98.6	BG-11 ^d^

^a^ Accession Number. ^b^ Best result in this genome. ^c^ Log2 fold change. ^d^ Detected exclusively in BG-11.

## Data Availability

The data presented in this study are available on request from the corresponding author.

## Supplementary Materials

The following are available online at https://www.mdpi.com/article/10.3390/microorganisms9081593/s1, Table S1: Morphological identification of the 63 enviromental samples, when available. Shown State and point of collection as well as inhibition time for β-glucosidase; Table S2: Proteins differently expressed present in at least two replicates of *Synechococcus* sp. GFB01.; Figure S1: Inhibitory activity of microalgae and cyanobacteria extracts. (A) Samples with longer inhibition for β-glucosidase on agar plate screening. Plates with microorganisms that inhibited the enzyme-substrate reaction for 60 min. (B) Samples with best result according to biological classification.
