# Supplementary material for: Glucosidase Inhibitors Screening in Microalgae and Cyanobacteria Isolated from the Amazon and Proteomic Analysis of Inhibitor Producing Synechococcus sp. GFB01
Source: Microorganisms. 2021 Jul 27;9(8):1593. doi: 10.3390/microorganisms9081593 (PMC8402191; doi:10.3390/microorganisms9081593)
Supplement: Supplementary file 1 [file microorganisms-09-01593-s001.zip › Table S2_ProteinID.pdf]

Table S2. Proteins differently expressed present in at least two replicates of *Synechococcus* sp. GFB01 grown on BG-11, with 1.5 g/L of $NaNO_3$ and BG-$11_{10\%N}$, with 0.15 g/L.

Accession Number	Molecular Weight	Protein ID	Log_2 FC[a]	KEGG Pathways[b]
		**Nitrogen Metabolism**		
WP_015168853	79 kDa	Glutamine synthetase	BG-$11_{10\%N}$[c]	Nitrogen metabolism, Glyoxylate and dicarboxylate metabolism, Arginine biosynthesis, Alanine, aspartate and glutamate metabolism
WP_011933362	79 kDa	Glutamine synthetase	BG-$11_{10\%N}$[c]	Nitrogen metabolism, Glyoxylate and dicarboxylate metabolism, Arginine biosynthesis, Alanine, aspartate and glutamate metabolism
WP_071800663	61 kDa	Urease subunit alpha	BG-11[d]	Purine metabolism, Arginine biosynthesis, Atrazine degradation
WP_071802070	57 kDa	Lysine--trna ligase	BG-11[d]	Aminoacyl-tRNA biosynthesis
WP_015125721	31 kDa	Nitrate transport ATP-binding subunits C and D	BG-11[d]	Purine metabolism, Thiamine metabolism
WP_038544987	53 kDa	Cluster of type I glutamate--ammonia ligase	BG-11[d]	Arginine biosynthesis, Nitrogen metabolism, Glyoxylate and dicarboxylate metabolism, Alanine, aspartate and glutamate metabolism
WP_011935713	52 kDa	Cluster of type I glutamate--ammonia ligase	BG-11[d]	Arginine biosynthesis, Nitrogen metabolism, Glyoxylate and dicarboxylate metabolism, Alanine, aspartate and glutamate metabolism
		**Carbohydrate metabolism**		
WP_038542455	37 kDa	Cluster of type I glyceraldehyde-3-phosphate dehydrogenase	2,85	Biosynthesis of antibiotics, Glycolysis / Gluconeogenesis, Carbon fixation in photosynthetic organisms
WP_006849626	52 kDa	Cluster of pyruvate kinase	1,48	Biosynthesis of antibiotics, Glycolysis / Gluconeogenesis, Carbon fixation in photosynthetic organisms, Pyruvate metabolism
WP_012305714	-	Enolase	0,5	Biosynthesis of antibiotics, Glycolysis / Gluconeogenesis, Carbon fixation in photosynthetic organisms
WP_071800536	45 kDa	Phosphopyruvate hydratase	0,15	Biosynthesis of antibiotics, Glycolysis / Gluconeogenesis, Methane metabolism

WP_071799845	38 kDa	Fructose-1,6-bisphosphate aldolase	-0,43	Biosynthesis of antibiotics, Glycolysis / Gluconeogenesis, Carbon fixation in photosynthetic organisms, Methane metabolism, Pentose phosphate pathway, Fructose and mannose metabolism
WP_038544582	63 kDa	Pyruvate kinase	-0,98	Biosynthesis of antibiotics, Glycolysis / Gluconeogenesis, Purine metabolism, Pyruvate metabolism
WP_011934446	37 kDa	Type I glyceraldehyde-3-phosphate dehydrogenase	-0,99	Biosynthesis of antibiotics, Glycolysis / Gluconeogenesis, Carbon fixation in photosynthetic organisms
WP_015125056	46 kDa	Phosphopyruvate hydratase	-1,46	Biosynthesis of antibiotics, Glycolysis / Gluconeogenesis, Methane metabolism
WP_038544004	39 kDa	Cluster of fructose-1,6-bisphosphate aldolase	-2,6	Biosynthesis of antibiotics, Glycolysis / Gluconeogenesis, Carbon fixation in photosynthetic organisms, Methane metabolism
WP_071802277	64 kDa	Pyruvate kinase	-2,94	Biosynthesis of antibiotics, Glycolysis / Gluconeogenesis, Purine metabolism, Pyruvate metabolism
WP_011933125	63 kDa	Pyruvate kinase	-3,41	Biosynthesis of antibiotics, Glycolysis / Gluconeogenesis, Purine metabolism, Pyruvate metabolism
WP_065712859	37 kDa	Type I glyceraldehyde-3-phosphate dehydrogenase	BG-11[d]	Biosynthesis of antibiotics, Carbon fixation in photosynthetic organisms, Glycolysis / Gluconeogenesis
WP_011935672	35 kDa	Fructose 1,6-bisphosphatase	BG-11[d]	Biosynthesis of antibiotics, Carbon fixation in photosynthetic organisms, Glycolysis / Gluconeogenesis, Pentose phosphate pathway, Methane metabolism, Fructose and mannose metabolism
WP_071800756	37 kDa	Cluster of type I glyceraldehyde-3-phosphate dehydrogenase	BG-11[d]	Biosynthesis of antibiotics, Carbon fixation in photosynthetic organisms, Glycolysis / Gluconeogenesis
WP_071800557	42 kDa	Phosphoglycerate kinase	BG-11[d]	Biosynthesis of antibiotics, Carbon fixation in photosynthetic organisms, Glycolysis / Gluconeogenesis
WP_015124780	37 kDa	Type I glyceraldehyde-3-phosphate dehydrogenase	BG-11[d]	Biosynthesis of antibiotics, Carbon fixation in photosynthetic organisms, Glycolysis / Gluconeogenesis
		**Stress response**		
WP_011378479	68 kDa	Cluster of chaperone protein dnak2	2,24	

WP_011936929	68 kDa	Cluster of molecular chaperone dnak	1,97	
WP_038546438	11 kDa	Co-chaperone groes	1,83	
WP_043737222	11 kDa	Co-chaperone groes	0,87	
WP_011936275	58 kDa	Cluster of molecular chaperone groel	0,16	
WP_071801034	24 kDa	Cluster of peroxidase	-0,55	Glutathione metabolism
WP_071799921	94 kDa	Cluster of ATP-dependent Clp protease ATP-binding subunit clpc	-0,71	
WP_038556864	11 kDa	Co-chaperone groes	-0,15	
WP_011933761	58 kDa	Cluster of molecular chaperone groel	-1,4	
WP_015124241	11 kDa	Co-chaperone groes	-3,56	
WP_043692066	12 kDa	Cluster of thiol reductase thioredoxin	-1,2	
WP_011243156	58 kDa	Molecular chaperone groel	BG-11[d]	
WP_071800033	57 kDa	Chaperonin grol	BG-11[d]	
WP_015169314	58 kDa	Molecular chaperone groel	BG-11[d]	
WP_015123389	57 kDa	Molecular chaperone groel	BG-11[d]	
WP_065715742	58 kDa	Cluster of chaperonin grol	BG-11[d]	
		**Photosynthesis and Carbon Fixation**		
WP_065714405	99 kDa	Cluster of photosystem I reaction center subunit X	$BG\text{-}11_{10\%N}$[c]	
WP_065714301	40 kDa	Cluster of coproporphyrinogen III oxidase	$BG\text{-}11_{10\%N}$[c]	Porphyrin and chlorophyll metabolism
WP_015122994	8 kDa	NAD(P)H-quinone oxidoreductase subunit O	$BG\text{-}11_{10\%N}$[c]	
WP_038543210	17 kDa	Phycocyanin subunit alpha	5,58	
WP_071801204	18 kDa	Allophycocyanin	2,48	
WP_042502838	29 kDa	Cluster of phycobilisome rod-core linker polypeptide cpcg	1,69	
WP_011936302	17 kDa	Allophycocyanin subunit alpha	1,42	
WP_011242807	17 kDa	Phycocyanin subunit alpha	1,37	
WP_043692601	11 kDa	Cluster of ferredoxin	1,18	

WP_015167845	51 kDa	ATP synthase subunit beta	1,02	Purine metabolism, Thiamine metabolism
WP_071801519	18 kDa	Cluster of phycocyanin subunit beta	0,83	
WP_043695027	17 kDa	Phycocyanin subunit alpha	0,79	
WP_071800804	9 kDa	Photosystem I subunit VII	0,72	
WP_051847317	45 kDa	Cluster of aspartate aminotransferase family protein	0,56	Porphyrin and chlorophyll metabolism
WP_011363412	-	Photosystem I reaction center subunit II (psad)	0,55	
WP_038546441	52 kDa	Cluster of ATP synthase subunit beta	0,48	Purine metabolism, Thiamine metabolism
WP_038546571	17 kDa	Cluster of allophycocyanin	0,31	
WP_071801197	54 kDa	Cluster of F0F1 ATP synthase subunit alpha	0	Purine metabolism, Thiamine metabolism
WP_038543910	43 kDa	Cluster of ferredoxin--NADP(+) reductase	-0,02	Photosynthesis
WP_071800963	16 kDa	Photosystem I reaction center subunit II	-0,2	
WP_015124682	53 kDa	Cluster of ribulose-bisphosphate carboxylase large subunit	-0,22	Biosynthesis of antibiotics, Carbon fixation in photosynthetic organisms, Glyoxylate and dicarboxylate metabolism
WP_038543749	53 kDa	Cluster of ribulose-bisphosphate carboxylase large subunit	-0,3	Biosynthesis of antibiotics, Carbon fixation in photosynthetic organisms, Glyoxylate and dicarboxylate metabolism
WP_006850103	-	Photosystem I iron-sulfur center	-0,46	
WP_011933546	51 kDa	Cluster of photosystem II 44 kda subunit reaction center protein	-0,61	
WP_071800981	32 kDa	Photosystem I reaction center subunit XII	-0,84	
WP_071802316	45 kDa	Glutamate-1-semialdehyde-2,1-aminomutase	-0,97	Porphyrin and chlorophyll metabolism
WP_043738506	17 kDa	Photosystem II protein psbq	-1,03	
WP_071801205	18 kDa	Cluster of allophycocyanin subunit beta	-1,33	
WP_038551076	17 kDa	Phycocyanin subunit alpha	-1,37	
WP_011936303	18 kDa	Allophycocyanin subunit beta	-1,46	
WP_006169870	-	Cluster of carbon dioxide-concentrating mechanism protein ccmk	-1,96	
WP_071801051	29 kDa	Phycobilisome rod-core linker polypeptide cpcg	-2,13	
WP_065713391	52 kDa	Cluster of F0F1 ATP synthase subunit beta	-2,5	Purine metabolism, Thiamine metabolism

WP_011936478	17 kDa	Phycocyanin subunit alpha	-3,29	
WP_038551544	21 kDa	NAD(P)H-quinone oxidoreductase subunit J	BG-11[d]	Oxidative phosphorylation
WP_038546567	105 kDa	Cell wall anchor protein	BG-11[d]	
WP_011936301	103 kDa	Anchor polypeptide LCM	BG-11[d]	
WP_011933917	107 kDa	Anchor polypeptide LCM	BG-11[d]	
WP_041430506	46 kDa	Aspartate aminotransferase family protein	BG-11[d]	Porphyrin and chlorophyll metabolism
WP_011936453	59 kDa	Cluster of phycobilisome linker polypeptide, C-phycoerythrin class I and II-associated	BG-11[d]	
WP_015167166	46 kDa	Aspartate aminotransferase family protein	BG-11[d]	Porphyrin and chlorophyll metabolism
WP_038543240	60 kDa	Phycobilisome linker polypeptide	BG-11[d]	
WP_006171988	8 kDa	Photosystem I reaction center subunit XII	BG-11[d]	
WP_011128093	18 kDa	Allophycocyanin subunit alpha	BG-11[d]	
WP_038543017	39 kDa	Photosystem II q(b) protein	BG-11[d]	
WP_011934836	57 kDa	Cluster of photosystem II chlorophyll-binding protein CP47	BG-11[d]	
WP_011936572	84 kDa	Cluster of photosystem I core protein psaa	BG-11[d]	
WP_011933748	19 kDa	Cluster of cytochrome b6-f complex iron-sulfur subunit	BG-11[d]	
WP_071800060	40 kDa	Cluster of magnesium chelatase atpase subunit I	BG-11[d]	Porphyrin and chlorophyll metabolism
WP_041428403	21 kDa	Carbon dioxide-concentrating protein ccmk	BG-11[d]	
WP_012307244	-	Cluster of allophycocyanin subunit beta-18	BG-11[d]	
WP_038557345	13 kDa	Cluster of (2Fe-2S)-binding protein	BG-11[d]	
WP_038547847	8 kDa	Photosystem I reaction center subunit IV	BG-11[d]	
WP_071820831	-	Cluster of cytochrome c-550	BG-11[d]	
WP_011243295	72 kDa	Transketolase	BG-11[d]	Biosynthesis of antibiotics, Carbon fixation in photosynthetic organisms, Pentose phosphate pathway, Biosynthesis of ansamycins
WP_011934548	72 kDa	Transketolase	BG-11[d]	Biosynthesis of antibiotics, Carbon fixation in photosynthetic organisms, Pentose phosphate pathway, Biosynthesis of

				ansamycins
		**Protein metabolism**		
WP_071801129	12 kDa	RNA-binding protein	BG-11$_{10\%N}$[c]	
WP_015125769	13 kDa	50S ribosomal protein L14	BG-11$_{10\%N}$[c]	
WP_015169097	45 kDa	Translation elongation factor Tu	5,14	Purine metabolism, Thiamine metabolism
WP_015123964	45 kDa	Translation elongation factor Tu	4,54	Purine metabolism, Thiamine metabolism
WP_011936540	13 kDa	Cluster of 50S ribosomal protein L14	1,07	
WP_011936583	75 kDa	Cluster of elongation factor G	0,94	Purine metabolism, Thiamine metabolism
WP_038543261	45 kDa	Cluster of methionine adenosyltransferase	0,89	Cysteine and methionine metabolism
WP_011933876	56 kDa	Cluster of phosphoglycerate dehydrogenase	0,17	Biosynthesis of antibiotics, Methane metabolism, Glycine, serine and threonine metabolism
WP_038543040	44 kDa	Cluster of translation elongation factor Tu	-0,26	Purine metabolism, Thiamine metabolism
WP_038555181	58 kDa	Cluster of dihydroxy-acid dehydratase	-0,76	Biosynthesis of antibiotics, Pantothenate and CoA biosynthesis, Valine, leucine and isoleucine biosynthesis
WP_038543120	16 kDa	Cluster of 50S ribosomal protein L15	-1,43	
WP_038545800	36 kDa	Cluster of ketol-acid reductoisomerase	BG-11[d]	Biosynthesis of antibiotic, Pantothenate and CoA biosynthesis, Valine, leucine and isoleucine biosynthesis
WP_038543141	27 kDa	Cluster of 30S ribosomal protein S3	BG-11[d]	
WP_038543044	18 kDa	Cluster of 30S ribosomal protein S7	BG-11[d]	
WP_071800543	13 kDa	Cluster of 50S ribosomal protein L7/L12	BG-11[d]	
WP_011933234	27 kDa	30S ribosomal protein S2	BG-11[d]	
WP_015123450	38 kDa	Cluster of 30S ribosomal protein S1	BG-11[d]	
WP_015168995	75 kDa	Molecular chaperone dnak	BG-11[d]	
WP_011936551	14 kDa	Cluster of 30S ribosomal protein S13	BG-11[d]	
WP_043695862	23 kDa	Cluster of 30S ribosomal protein S4	BG-11[d]	
WP_011936081	17 kDa	Cluster of 50S ribosomal protein L19	BG-11[d]	
WP_038543146	32 kDa	Cluster of 50S ribosomal protein L2	BG-11[d]	

WP_006850182	14 kDa	30S ribosomal protein S11	BG-11[d]	
WP_043694882	23 kDa	Cluster of 30S ribosomal protein S5	BG-11[d]	
		**Cell Division and Nucleic Acid Synthesis**		
WP_011936170	68 kDa	Cell division protein ftsh	-1,3	Purine metabolism, Thiamine metabolism
WP_041428475	68 kDa	Cluster of abrb family transcriptional regulator	2,48	
WP_071799987	39 kDa	Cluster of DNA-directed RNA polymerase subunit gamma	0,9	
WP_011934961	78 kDa	Cluster of nucleoside-diphosphate kinase	0,67	Drug metabolism, Pyrimidine metabolism
WP_011935819	69 kDa	Cluster of polyribonucleotide nucleotidyltransferase	0,07	
WP_071801860	67 kDa	Cluster of DNA-directed RNA polymerase subunit beta	-0,13	
WP_042503472	63 kDa	Cluster of transcription termination factor nusa	-0,4	
WP_011377820	69 kDa	Cluster of DNA-directed RNA polymerase subunit beta	-1,82	
WP_011935906	71 kDa	RNA-binding protein	-1,88	
WP_043691164	65 kDa	RNA-binding protein	-2,14	
WP_051834421	63 kDa	Cell division protein ftsh	BG-11[d]	Purine metabolism, Thiamide metabolism
WP_011933123	69 kDa	Cluster of cell division protein ftsz	BG-11[d]	Purine metabolism
WP_065712803	68 kDa	Polyribonucleotide nucleotidyltransferase	BG-11[d]	
WP_071801461	69 kDa	Cell division protein ftsh	BG-11[d]	Purine metabolism, Thiamine metabolism
WP_011936440	40 kDa	Cell division protein ftsh	BG-11[d]	Purine metabolism, Thiamine metabolism
WP_015125867	104 kDa	Cell division protein ftsh	BG-11[d]	Purine metabolism, Thiamine metabolism
WP_065713676	138 kDa	Cell division protein ftsh	BG-11[d]	Purine metabolism, Thiamine metabolism
WP_038548030	13 kDa	Cell division protein ftsh	BG-11[d]	Purine metabolism, Thiamine metabolism
WP_015123971	71 kDa	Cell division protein ftsh	BG-11[d]	Purine metabolism, Thiamine metabolism
WP_038552400	16 kDa	Cell division protein ftsh	BG-11[d]	Purine metabolism, Thiamine metabolism
WP_043695309	78 kDa	Cell division protein ftsh	BG-11[d]	Purine metabolism, Thiamine metabolism
WP_071801112	122 kDa	Cluster of cell division protein ftsh	BG-11[d]	Purine metabolism, Thiamine metabolism
WP_038546210	55 kDa	Cell division protein ftsh	BG-11[d]	Purine metabolism, Thiamine metabolism

WP_015123972	124 kDa	Cluster of DNA-directed RNA polymerase subunit beta''	BG-11[d]
WP_038544302	11 kDa	Cluster of transcription termination/antitermination factor nusg	BG-11[d]
WP_011935496	10 kDa	DNA-directed RNA polymerase subunit beta''	BG-11[d]
WP_065714415	145 kDa	Cluster of DNA-directed RNA polymerase subunit beta'	BG-11[d]
WP_038547297	25 kDa	DNA-directed RNA polymerase subunit beta'	BG-11[d]
WP_071801114	148 kDa	Diguanylate cyclase	BG-11[d]
WP_038546187	148 kDa	Abrb family transcriptional regulator	BG-11[d]
WP_011936371	148 kDa	Hypothetical protein	BG-11$_{10\%N}$[c]
WP_071801584	18 kDa	DNA repair atpase	BG-11$_{10\%N}$[c]
WP_038545490	13 kDa	Cluster of chromosome segregation protein SMC	BG-11$_{10\%N}$[c]
		**Transport and Morphology**	
WP_041428323	46 kDa	Hemolysin activation protein	BG-11$_{10\%N}$[c]
WP_065710894	17 kDa	Hypothetical protein	BG-11$_{10\%N}$[c]
WP_071801685	56 kDa	Cluster of porin	BG-11$_{10\%N}$[c]
WP_071800858	34 kDa	Phosphate ABC transporter substrate-binding protein psts	-0,11
WP_011243524	36 kDa	Rod shape-determining protein	BG-11[d]
WP_011129042	62 kDa	Cluster of RND transporter	BG-11[d]
WP_038547629	37 kDa	Cluster of rod shape-determining protein	BG-11[d]
WP_043692566	80 kDa	Membrane protein	BG-11[d]
WP_011934123	68 kDa	Cluster of porin	BG-11[d]
		**Hipothetical and Other Proteins**	
WP_011932576	37 kDa	Hypothetical protein	BG-11$_{10\%N}$[c]
WP_041428492	16 kDa	Peptidyl-prolyl cis-trans isomerase	4,78
WP_043738095	15 kDa	Peptidyl-prolyl cis-trans isomerase	4,51

WP_071801041	23 kDa	Protein phosphatase	0,17
ID_40449	-	Identified yet undeclared protein #40449 in proteinlynx output	-0,3
WP_038557135	16 kDa	Peptidyl-prolyl cis-trans isomerase	-4,26
WP_043736997	51 kDa	Hypothetical protein	BG-11[d]
WP_065715110	53 kDa	Cluster of hypothetical protein	BG-11[d]
ID_42961	-	Cluster of Identified yet undeclared protein #42961 in proteinlynx output	BG-11[d]
WP_043691076	63 kDa	Signal protein PDZ	BG-11[d]
ID_15159	-	Identified yet undeclared protein #15159 in proteinlynx output	BG-11[d]

[a] Log_2 Fold change with ≥ 1 for more abundant proteins under nutritional stress, and ≤ -1 for less abundant proteins.
[b] KEGG pathway according to automatic annotation on Blast2GO
[c] Present exclusively in $BG\text{-}11_{10\%N}$
[d] Present exclusively in BG-11
