# Supplementary material for: Glucosidase Inhibitors Screening in Microalgae and Cyanobacteria Isolated from the Amazon and Proteomic Analysis of Inhibitor Producing Synechococcus sp. GFB01
Source: Microorganisms. 2021 Jul 27;9(8):1593. doi: 10.3390/microorganisms9081593 (PMC8402191; doi:10.3390/microorganisms9081593)
Supplement: Supplementary file 1 [file microorganisms-09-01593-s001.zip › Table_S1_samplesLocation.pdf]

Supplementary Materials

Table S1. Morphological identification of the 63 enviromental samples, when available. State and point of collection as well as inhibition time for β-glucosidase are shown.

Sample	Morphologycal ID	State	Colection Site	Inhibition Time (minutes)
**GFB01**	*Synechococcus sp.*[a]	Amapá	Lagoa dos Índios	60 (Crude and Methanolic extracts)
**A1**	*Chlorella sp.*	Tocantins	Ilha Verde (A)	60
**B3**	*Chlorella sp.*	Tocantins	Palma's shore	60
**B4**	*Chlorella sp.*	Tocantins	Palma's shore	60
**B6**	*Chlorella sp.*	Tocantins	Lagoa da Confusão	60
**M**	*Monoraphidium sp.*	Amapá	Lagoa dos Índios	60
**P29**	*Limnothrix sp.*	Amapá	Lagoa dos Índios	60
**P38**	*Limnothrix sp.*	Amapá	Igarapé da Fortaleza	60
**A10**	-	Tocantins	Palma's shore	30
**A11**	-	Tocantins	Porto Nacional (rocks)	30
**A12**	-	Tocantins	Rice plantation	30
**A16**	-	Tocantins	Rice plantation	30
**A17**	-	Tocantins	Porto Nacional	30
**A18**	-	Tocantins	Porto Nacional	30
**A19**	-	Tocantins	Porto Nacional	30
**A20**	-	Tocantins	Ilha Verde (B)	30
**A27**	-	Tocantins	Lagoa da Confusão	30
**B1**	-	Tocantins	Porto Nacional (rocks)	30
**B2**	-	Tocantins	Lagoa da Confusão	30
**R13**	*Limnothrix sp.*	Amapá	Igarapé da Fortaleza	30
**A13**	-	Tocantins	Lagoa da Confusão	15
**A14**	-	Tocantins	Ilha Verde (A)	15
**A15**	-	Tocantins	Rod. TO010, km22	15
**A21**	-	Tocantins	Ilha Verde (B)	15
**A22**	-	Tocantins	Lagoa da Confusão	15
**A23**	-	Tocantins	Lagoa da Confusão	15
**B7**	-	Tocantins	Lagoa da Confusão	15
**B8**	-	Tocantins	Lagoa da Confusão	15
**B9**	-	Tocantins	Lagoa da Confusão	15
**B10**	-	Tocantins	Rod. TO010, km22	15
**B11**	-	Tocantins	Rod. TO010, km22	15
**B12**	-	Tocantins	Ilha Verde (B)	15
**B14**	-	Tocantins	Rice plantation	15
**B15**	-	Tocantins	Rice plantation	15
**B17**	-	Tocantins	Palma's shore	15
**B18**	-	Tocantins	Palma's shore	15
**B19**	-	Tocantins	Palma's shore	15
**B20**	-	Tocantins	Lagoa da Confusão	15
**B22**	-	Tocantins	Porto Nacional	15
**B23**	*Stigeoclonium sp.*	Amapá	Water puddle	15
**B24**	-	Tocantins	Porto Nacional	15

**P**	*Planktothrix sp.*	Amapá	Lagoa dos Índios	15
**P15**	*Nostoc sp.*	Amapá	Lagoa dos Índios	15
**Q17**	*Limnothrix sp.*	Amapá	Igarapé da Fortaleza	15
**Q36**	*Limnothrix sp.*	Amapá	Igarapé da Fortaleza	15
**B16**	-	Tocantins	Ilha Verde (A)	10
**B5**	-	Tocantins	Ilha Verde (B)	10
**A2**	-	Tocantins	Ilha Verde (A)	10
**A6**	-	Tocantins	Rod. TO010, km22	10
**A7**	-	Tocantins	Ilha Verde (B)	10
**A9**	-	Tocantins	Ilha Verde (B)	10
**A24**	-	Tocantins	Lagoa da Confusão	10
**A25**	-	Tocantins	Rice plantation	10
**B13**	-	Tocantins	Lagoa da Confusão	10
**B21**	-	Tocantins	Ilha Verde (A)	10
**G**	*Limnothrix sp.*	Amapá	Lagoa dos Índios	10
**Q20**	-	Amapá	Lagoa dos Índios	10
**Q29**	*Merismopedia sp.*	Amapá	Igarapé da Fortaleza	10
**Q30**	*Limnothrix sp.*	Amapá	Igarapé da Fortaleza	10
**Q33**	*Limnothrix sp.*	Amapá	Lagoa dos índios	10
**Q35**	*Merismopedia sp.*	Amapá	Lagoa dos Índios	10
**R11**	*Limnothrix sp.*	Amapá	Igarapé da Fortaleza	10
**A8**	-	Tocantins	Ilha Verde (A)	5

[a]16s mRNA gene compatible with the genus *Synechococcus* (98% identity)
