# Supplementary figures and images for: Glucosidase Inhibitors Screening in Microalgae and Cyanobacteria Isolated from the Amazon and Proteomic Analysis of Inhibitor Producing Synechococcus sp. GFB01
Source: Microorganisms. 2021 Jul 27;9(8):1593. doi: 10.3390/microorganisms9081593 (PMC8402191; doi:10.3390/microorganisms9081593)

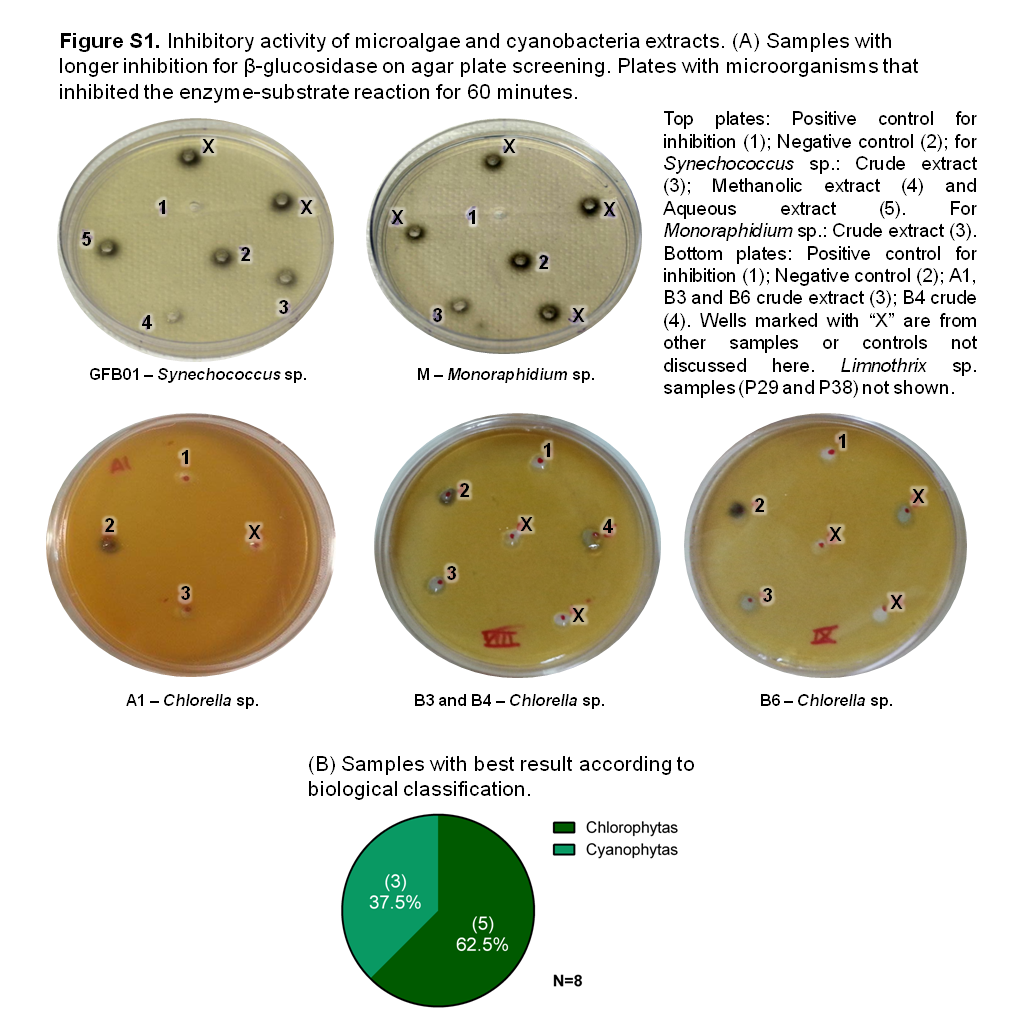

Supplement: Supplementary file 1 [file microorganisms-09-01593-s001.zip › FigureS1.png]
